# Supplementary figures and images for: Diverse neuronal activity patterns contribute to the control of distraction in the prefrontal and parietal cortex
Source: PLoS Biol. 2025 Jan 27;23(1):e3003008. doi: 10.1371/journal.pbio.3003008 (PMC11801722; doi:10.1371/journal.pbio.3003008)

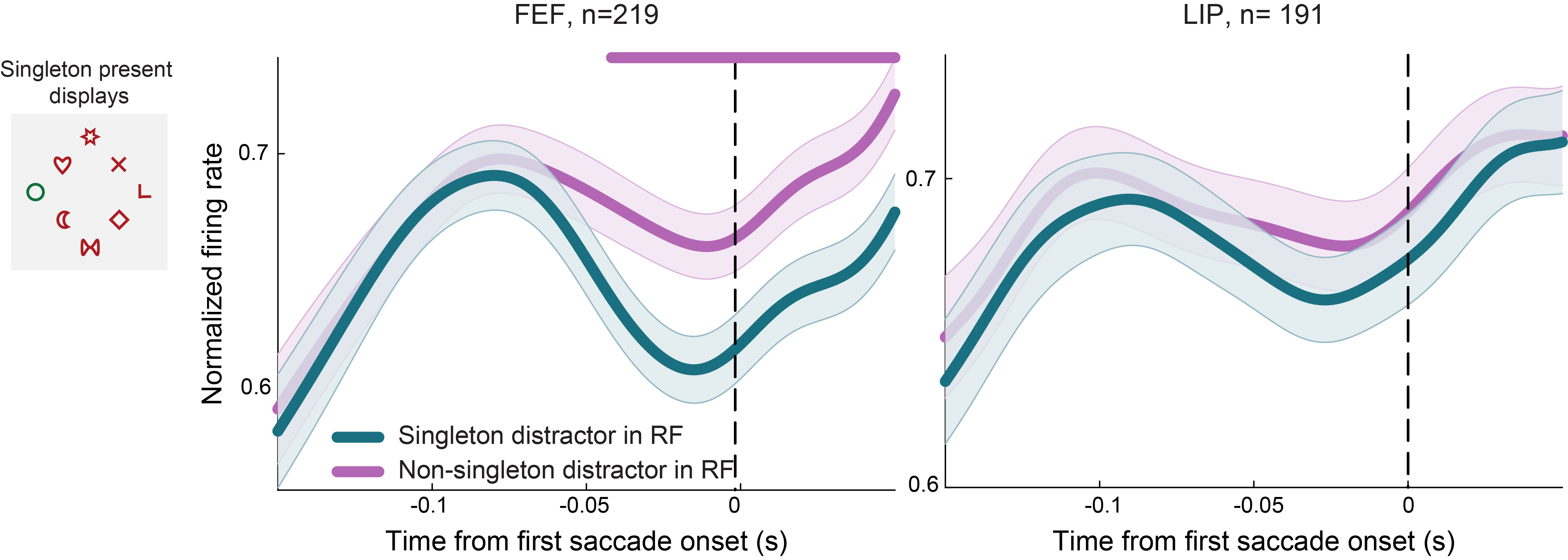

Supplement: S1 Fig — Responses were calculated in singleton present displays (example shown on the left). Normalized firing rates in singleton present displays with the singleton (green) and non-singleton (magenta) distractor in the RF, in FEF (left) and LIP (right). Error bars (shaded area around each line) represent ±SEM. The horizontal line at the top of each graph indicates periods with significant differences between the 2 conditions (permutation test, p < 0.05). Source data are available at https://zenodo.org/records/14577123. (TIF) [file pbio.3003008.s001.tif]

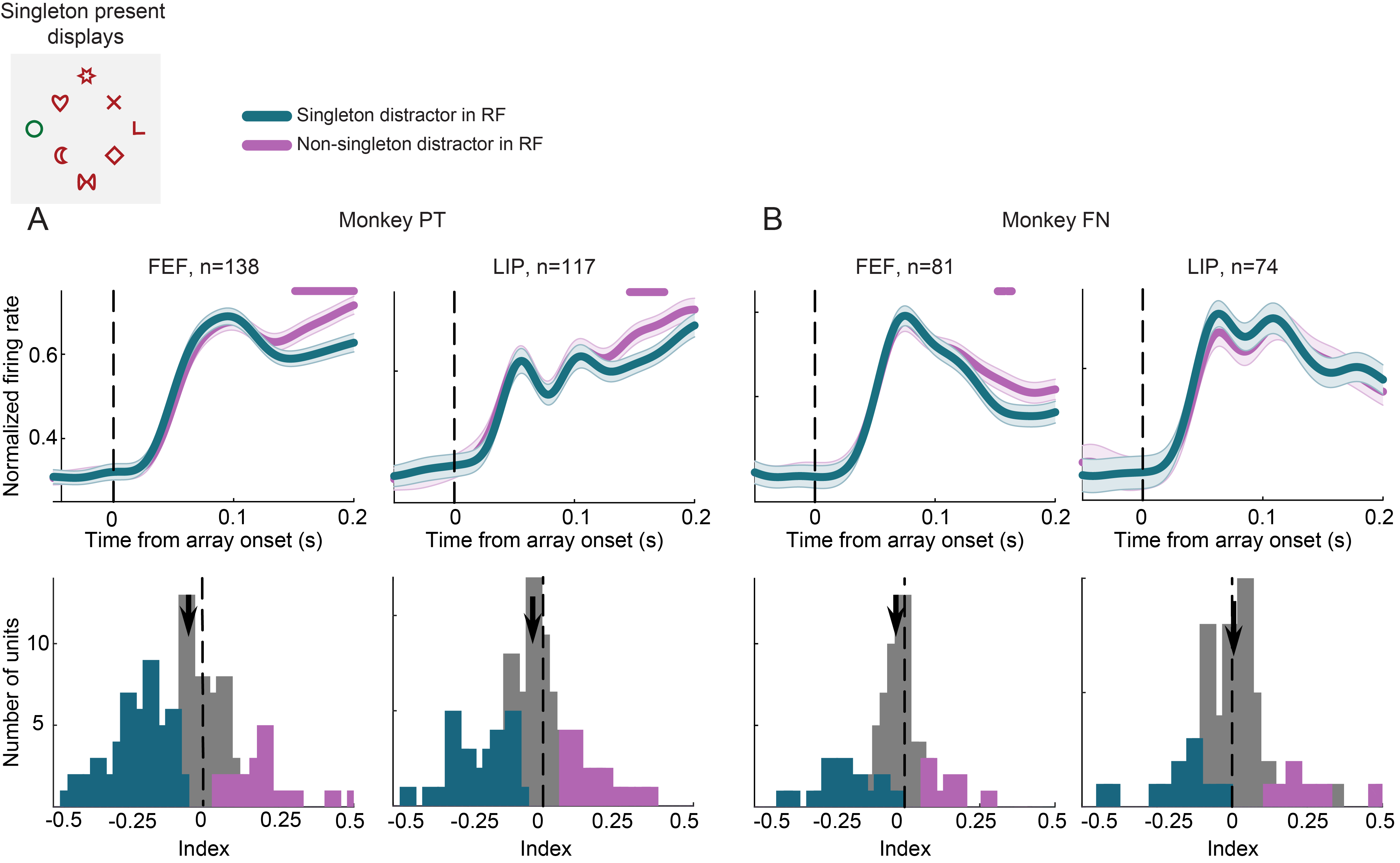

Supplement: S2 Fig — Responses in singleton and non-singleton distractor in RF were contrasted in singleton present displays (example display shown on top left). (A) Data for monkey PT. Top panels show normalized population average firing rates aligned to array onset in singleton present displays, with the singleton (green) and a non-singleton (magenta) distractor in the RF, in FEF (left) and LIP (right). Error bars (shaded area around each line) represent ±SEM. The horizontal line at the top of each graph indicates periods with significant differences between the 2 conditions (permutation test, p < 0.05). Bottom panels show distribution of modulation indices quantifying the difference between responses to the singleton and non-singleton distractors (150–200 ms following array onset) at the level of individual units in FEF (left) and LIP (right). Colored bars correspond to units with significant suppression (green) or enhancement (magenta) of responses for the singleton distractor (two-sample t test, p < 0.05). Arrows indicate the median of each distribution (FEF: −0.05; p < 0.001, LIP: −0.035, p < 0.01; Wilcoxon rank-sum test). (B) Data for monkey FN. Same conventions as in (A). The medians of each distribution are FEF: −0.03; p < 0.01, LIP: 0.005, p = 0.8; Wilcoxon rank-sum test. Source data are available at https://zenodo.org/records/14577123. (TIF) [file pbio.3003008.s002.tif]

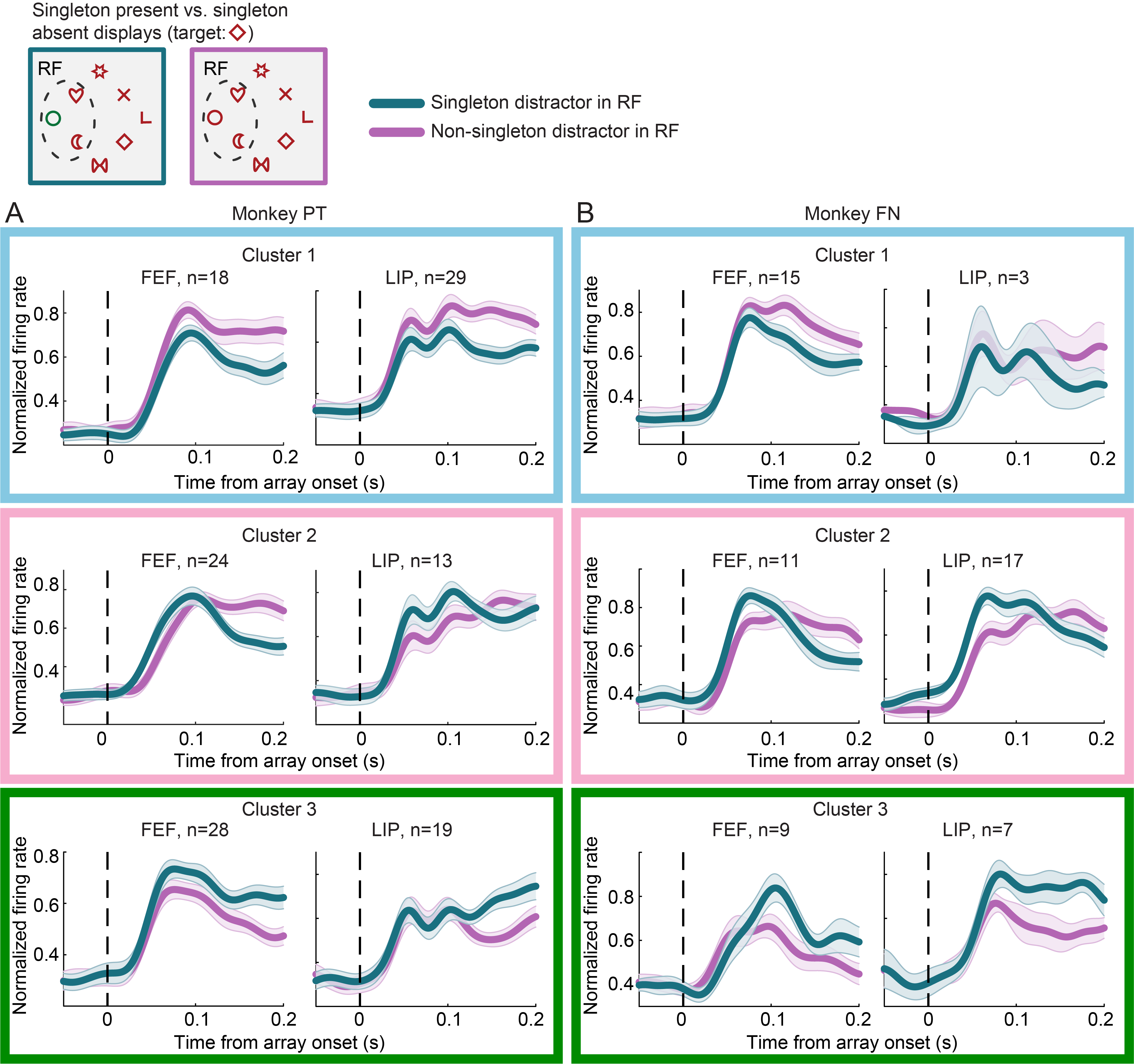

Supplement: S3 Fig — Responses in singleton present and singleton absent displays (example displays shown on top left) were contrasted. (A) The 3 clusters identified by the PhenoGraph algorithm for (A) monkey PT and (B) monkey FN. Graphs show average normalized firing rates in singleton (green) and non-singleton (magenta) in the RF trials. Error bars (shaded area around each line) represent ±SEM. Source data are available at https://zenodo.org/records/14577123. (TIF) [file pbio.3003008.s003.tif]

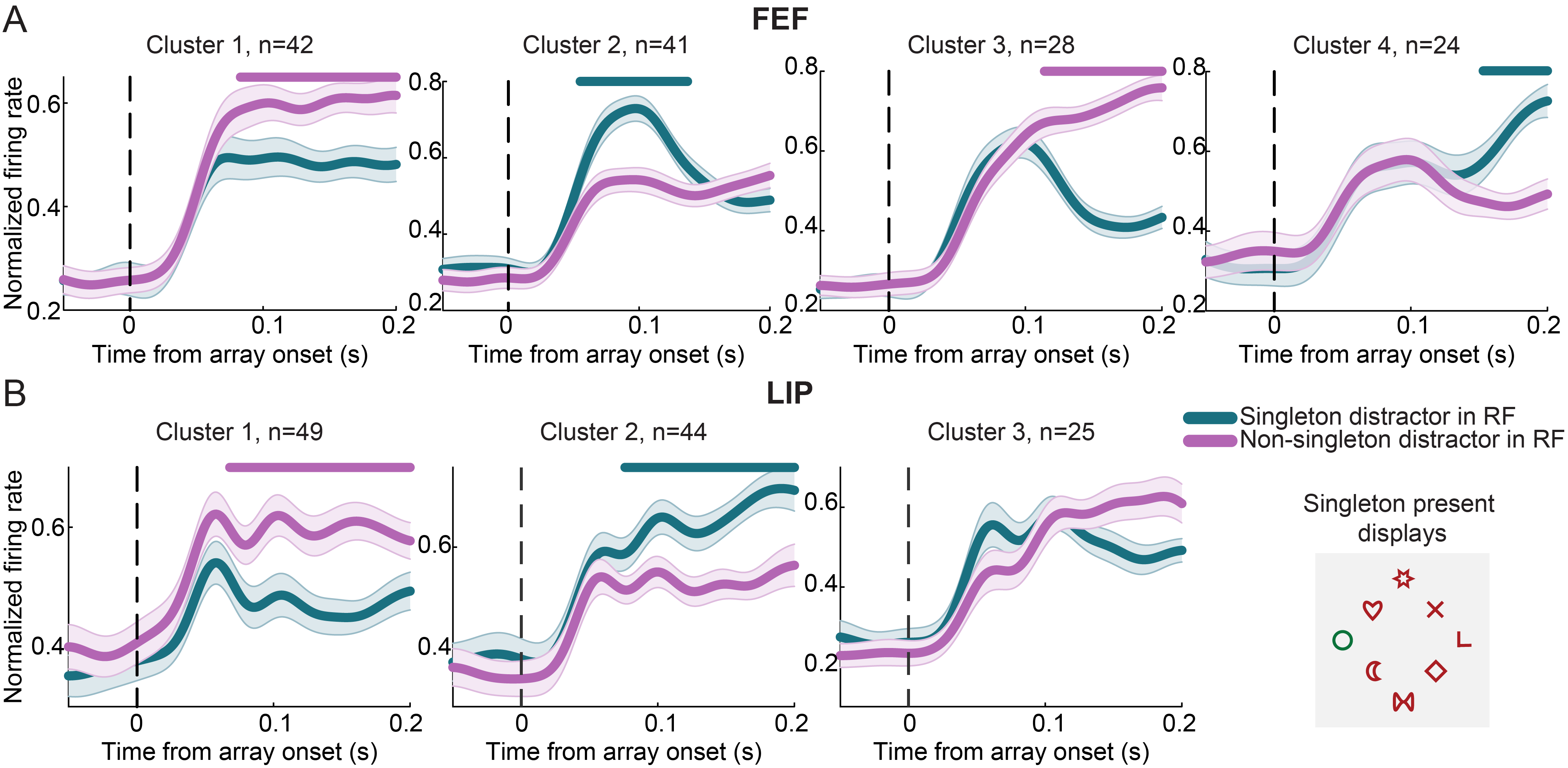

Supplement: S4 Fig — Responses were calculated from singleton-present displays (example shown on the bottom right). (A) The PhenoGraph algorithm identified 4 FEF subpopulations. Two of these subpopulations exhibited singleton suppression (clusters 1 and 3), one subpopulation showed singleton enhancement (cluster 4), and one exhibited an early enhancement followed by a later suppression (cluster 2). Panels show average firing rates in singleton (green) and non-singleton in RF (magenta) trials. Horizontal line at the top indicates periods with significant differences between the 2 conditions (permutation test, p < 0.05). (B) The clustering algorithm identified 3 LIP subpopulations. One exhibited singleton suppression (cluster 1), a second one showed singleton enhancement (cluster 2), and a third one an early enhancement followed by a subsequent suppression (cluster 3). Source data are available at https://zenodo.org/records/14577123. (TIF) [file pbio.3003008.s004.tif]

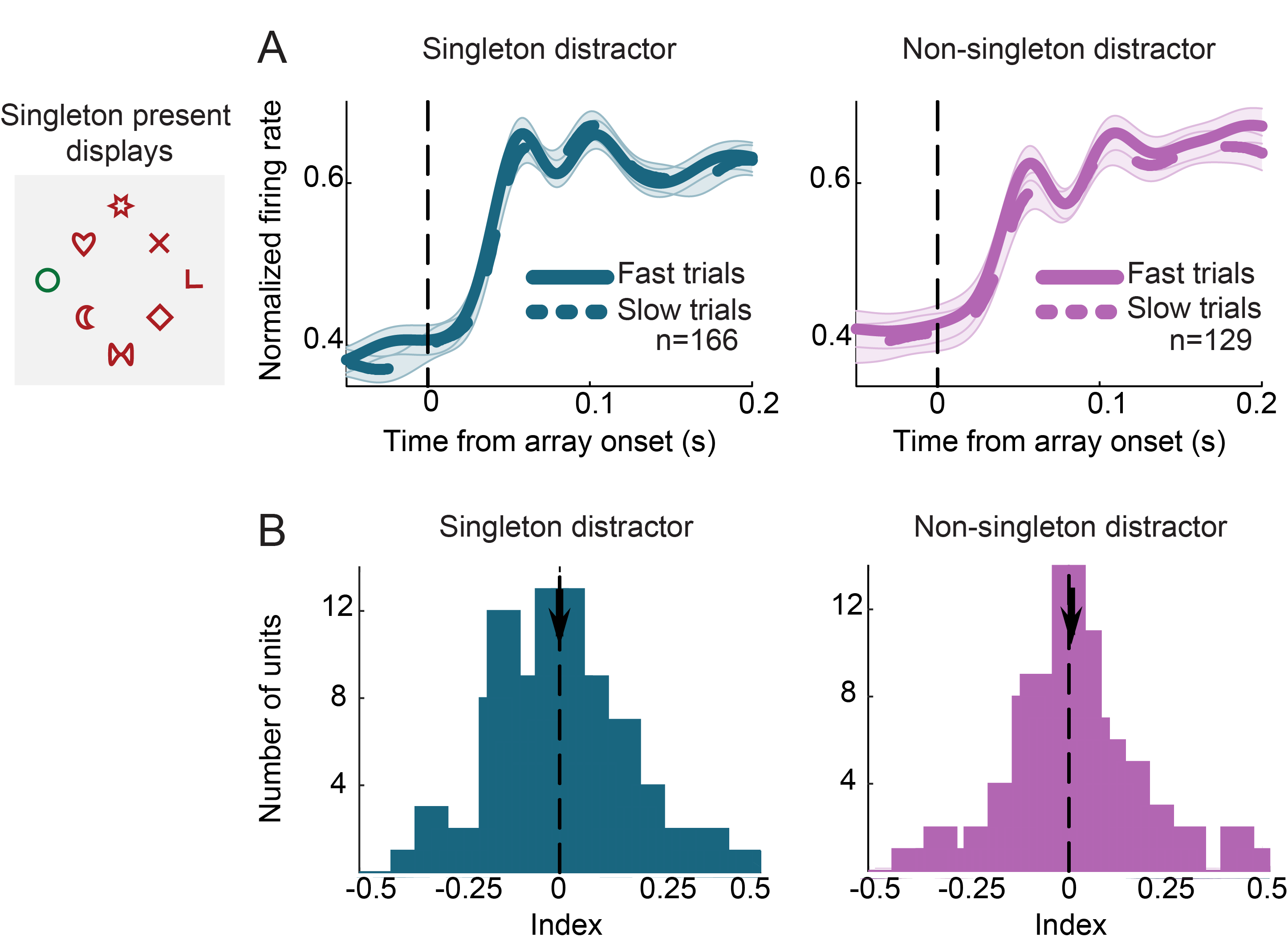

Supplement: S5 Fig — Responses were calculated in singleton present displays (example shown on the top left). (A) LIP responses to singleton (left) and non-singleton (right) distractor in RF in fast (solid line) and slow trials (dashed line). (B) Modulation indices quantifying the difference between fast and slow trials at the level of individual LIP units for singleton (left) and non-singleton (right) distractors in the RF. Arrows indicate the median of each distribution. Source data are available at https://zenodo.org/records/14577123. (TIF) [file pbio.3003008.s005.tif]

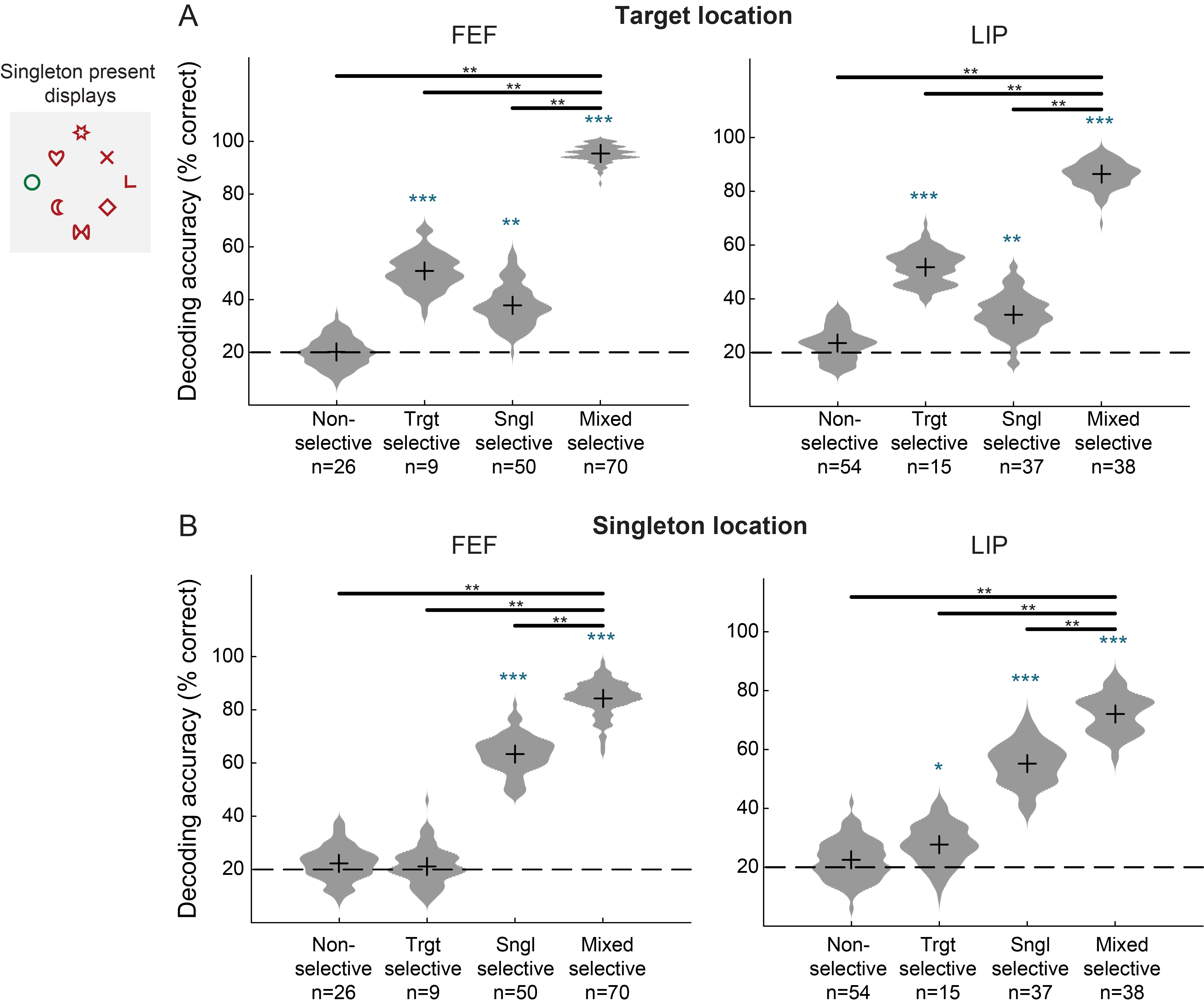

Supplement: S6 Fig — Responses were calculated in singleton present displays (example shown on top left). (A) Decoding of target location from FEF (left) and LIP (right) activity in the 150–200 ms interval after array onset. Violin plots show the distribution of decoding accuracies calculated over 50 resamples across different trials. The dashed horizontal line indicates chance accuracy. The 3 left hemifield and 2 vertical meridian locations were considered, so chance was at 20%. Crosses indicate the mean of each distribution. Blue stars above each distribution indicate significant accuracy relative to chance (permutation test). Stars above horizontal bars indicate difference between areas (permutation test). (B) Same for singleton location. Source data are available at https://zenodo.org/records/14577123. (TIF) [file pbio.3003008.s006.tif]

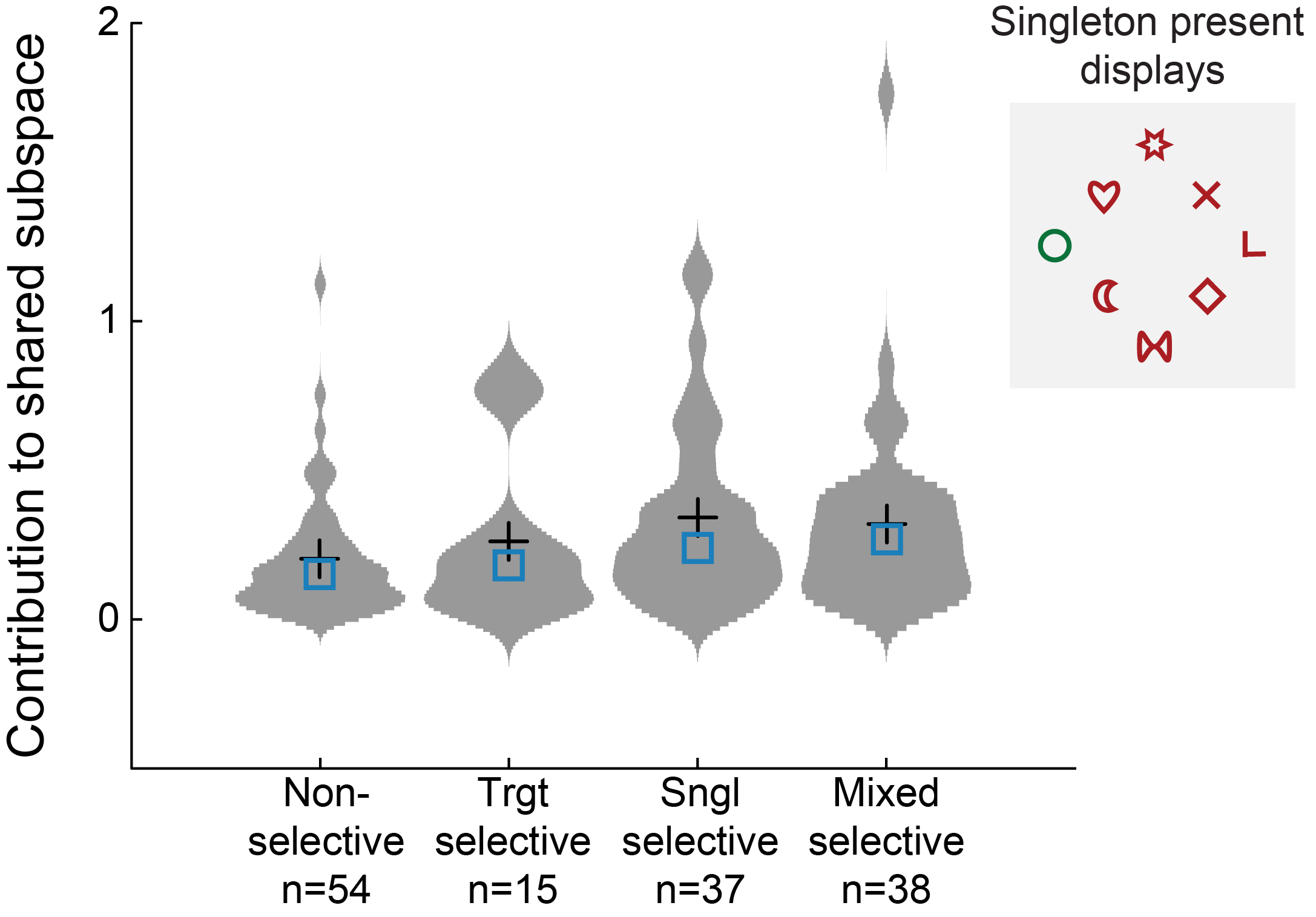

Supplement: S7 Fig — Violin plots show the distribution of weights contributing to the shared subspace for each category. Crosses represent the mean of each distribution and boxes the median. Data were obtained from the singleton present displays (shown on top right). Source data and relevant code are available at https://zenodo.org/records/14577123. (TIF) [file pbio.3003008.s007.tif]
