# Supplementary material for: Diverse neuronal activity patterns contribute to the control of distraction in the prefrontal and parietal cortex
Source: PLoS Biol. 2025 Jan 27;23(1):e3003008. doi: 10.1371/journal.pbio.3003008 (PMC11801722; doi:10.1371/journal.pbio.3003008)
Supplement: Attachment — Submitted filename: Response to Reviewers_final.docx [file pbio.3003008.s008.docx]

We thank the reviewers for their detailed, encouraging and insightful comments on the manuscript. We have revised the manuscript in response to the reviewers' comments and believe that the manuscript has improved significantly as a result. We address the points raised by the reviewers below.

*Reviewer #1: This is an interesting article examining how neurons in the frontal and parietal lobe represent task relevant and irrelevant stimuli. The frontal eye fields (FEF) and the lateral intraparietal area (LIP) have been thought to represent salient stimuli and filter distractors, with similar magnitude and time course. Recent results in the visual cortex, and technical developments in the analysis of neural population data have prompted the authors of this study to reconsider the question. They therefore have trained monkeys to perform a very difficult behavioral task which involves the selection of stimuli based on shape and color. Their results reveal a second population of neurons, whose activity is enhanced by the distractor, and systematic differences between the two areas. Furthermore, they show that representation of targets and salient distractors occupy a shared subspace. The results are novel and interesting and make a valuable addition to this literature. The manuscript is written clearly and is accessible to a wide audience, despite the highly technical nature of some results and analyses. There are some issues however that need to be addressed before the manuscript can be published.*

*1. The authors begin the presentation of their results with the comparison of the distractor suppression between areas. This result is probably the weakest of all the effects presented in the paper. Both FEF and LIP exhibit a pattern of singleton distractor suppression that is qualitatively similar. The magnitude of the FEF population suppression in figure 2B-left seems very small and the distribution of modulation indexes between the two areas in figure 2B-left and -right almost identical. Assuming that the reported statistics are correct as reported, the authors should include effect sizes and provide some intuition of how the comparison between the two samples that look so similar reached significance. In any case, toning down the presentation of this result would probably help the paper: in both areas* *there are neurons for which activity of the singleton distractor is suppressed - this is what can be unequivocally gleaned from this analysis.*

**Response:** The reviewer is right that the two distributions in Fig 2B look quite similar and that there are neurons in both areas whose activity for the singleton distractor is suppressed. The reviewer’s comment prompted us to explore in more detail possible reasons that contributed to a significant difference between the two distributions. We found a significantly larger proportion of FEF units with significant suppression relative to LIP (37% in FEF vs. 25% in LIP; p<0.01, chi-squared test). By contrast, the percentage of units with significant enhancement in the two areas was not significantly different (13.7% in FEF vs. 14.7% in LIP; p=0.8, chi-squared test). These results have been added to the relevant section in the manuscript. Moreover, following the reviewer’s suggestion, we now include effect sizes in the main text, use a more appropriate non-parametric test, and report the exact p-value (which shows marginal significance) when comparing the two distributions in Fig 2B. Finally, to tone down the conclusion based on the comparison between areas, as the reviewer suggested, we have now rephrased the title of the relevant section in the Results from “Singleton distractor is more strongly suppressed in FEF relative to LIP” to “Singleton distractor suppression is evident in both FEF and LIP” and rephrased the abstract, several parts of the relevant results section and parts of the Discussion accordingly, to shift the emphasis to the fact that there are units in both areas whose activity for the singleton distractor is suppressed.

*2. The latency of the effect could have been better explored. As the authors are aware, it has been proposed that signals of bottom-up attention appear earlier in LIP than FEF, whereas top-down attention follows the reverse sequence (an idea championed by reference 43). Compelling evidence has been presented since that the former is not true. The authors have the opportunity to disprove the latter claim as well, as their paradigm is a prototypical top-down attention task that requires subject to actively ignore bottom-up signals.*

**Response:** We thank the reviewer for this suggestion. In line with the previous comment, we found it more appropriate to examine latencies at the level of individual subpopulations (Fig 4), rather than in the entire population, where the heterogeneity of response profiles would obscure the latency of the effects. This analysis was carried out in the original manuscript and results were reported in the section “Distinct neuronal subpopulations manifest unique selectivity profiles” Consistent with the reviewer’s comment about the onset of bottom-up signals, we reported that in Cluster 2, which showed the earliest singleton enhancement across the three clusters, the enhancement effect occurred at similar times in the two areas disproving the idea that bottom-up attention signals appear earlier in LIP. This was also acknowledged in the Discussion citing relevant literature: “Nevertheless, we were able to compare the latency of the early singleton enhancement effects across FEF and LIP, and found that these occurred at similar times in the two areas. This is in line with a previous study, which showed that in monkeys trained to detect a salient singleton, prefrontal and parietal neurons show significant enhancements in activity at similar latencies (Katsuki & Constantinidis, 2012).” To contrast this finding with earlier reports, as the reviewer suggests, we have now added “…contrary to earlier accounts positing that bottom-up attention signals arise earlier in the parietal cortex relative to the PFC (Buschman & Miller, 2007)”.

Accordingly, to investigate the relative onset of the top-down signal, we focused on the suppression effect of Cluster 1 and Cluster 2. In the second paragraph of the Results section “Distinct neuronal subpopulations manifest unique selectivity profiles” we reported that in both cases the reduction occurred significantly earlier in FEF than LIP (p<0.01, permutation test). Therefore, based on these data, we cannot disprove that the top-down distractor suppression signal emerges earlier in the FEF.

*3. There is no discussion of results from the two individual monkeys in either the first or subsequent result sections. Some confirmation of the main findings of the study across both individuals would strengthen the paper.*

**Response:** We regret this omission, and we thank the reviewer for pointing this out. We now include results from individual monkeys as Supplementary Figures (Fig S2 and S3).

*4. Page 6, last sentence of the last complete paragraph: this point was not clear. Was the salient distractor suppression not evident or decodable for FEF?*

*[Revision along the lines suggested in point 1 may make this comment moot].*

**Response:** Following the reviewer’s suggestion in point 1, we have now revised this sentence to emphasize the existence of neurons in both areas for which the activity of the singleton distractor is suppressed. The sentence now reads *“*Overall, we found a larger proportion of units with significant suppression in the FEF, and higher overall decoding accuracies compared to LIP. However, our results show that neurons whose activity for the singleton distractor is significantly suppressed or enhanced exist in both areas, indicating that specific neuronal subpopulations carry the relevant information.”

*5. Describing a color as "petrol" may not be clear for all readers.*

**Response:** We thank the reviewer for pointing this out. We have modified the color and now refer to it as green.

*6. The authors include units in the selectivity analysis of page 9 if they exhibited significant t-values for 10 consecutive 1-ms bins. The choice of bin duration seems very short. How many trials were available to provide such a level of precision?*

**Response:** As outlined in Materials and Methods, we generated spike density functions by convolving spikes with a Gaussian filter (sigma=10 ms) and included units with at least 10 trials, although, on average, there were approximately 35 trials available for each unit in each condition. From these convolved responses, we calculated t-values over time that were, overall, robust and in most cases exceeded the 10 ms significance threshold. Following the reviewer’s comment, in the Materials and Methods, we now clarify that all firing rate analyses were carried out using the convolved signals. If the use of the convolved signal raises concerns about the accuracy of the latency calculation, we note in the Discussion that the reported latencies do not necessarily reflect the absolute latency of the effects, but rather that we aimed to compare their relative onset in the two areas.

*7. Page 11, first sentence. The authors do not quite show such a strong, inverse relationship. This statement should be rephrased.*

**Response:** Following the reviewer’s comment we have now included effect sizes (which appear large) and rephrased the relevant statement that now reads “Overall, our findings indicate a correlation between distractor suppression in the FEF and the time needed to locate the target. Therefore, suppression of salient stimuli implemented in the FEF seems to facilitate efficient target selection.”

*8. Findings of mixed selectivity were very interesting. In recent years, a lot of attention has been devoted particularly to nonlinear mixed selectivity i.e. selectivity for a stimulus dimension that changes nonlinearly across different contexts. It would be interesting for the authors to further break down their results in units that show linear and nonlinear mixed selectivity.*

**Response:** We thank the reviewer for raising this point. This would indeed be an interesting direction in our analysis. A typical approach to address this question would be to perform a 2-way ANOVA and identify nonlinear mixed selective neurons as those with a significant interaction term between factors (commonly seen as a switch in selectivity across contexts).

Following this approach, our analysis showed that units selective for both the target and singleton distractor had a significant interaction term, suggesting that units in our FEF and LIP populations changed their spatial selectivity for targets and salient distractors. Given that distinct subpopulations showed different activity patterns (enhancement and suppression effects for the salient distractor), it is hard to translate this change in spatial selectivity into a single mechanism. For example, a change in selectivity for the salient distractor could be due to maximal suppression for the preferred target position and less suppression for other positions (for neurons with mainly suppressive effects), or to an enhancement of activity for the salient distractor for a position different than the preferred target position (for neurons with enhancement effects mainly). Thus, to address the reviewer’s comment it would be useful to explore spatial selectivity for targets and salient distractors for the different subpopulations identified in Fig 4. Unfortunately, limitations in our study (e.g., the number of trials that were available for each unit per location and context, the small number of units in each subpopulation etc.), do not allow us to examine how different subpopulations (those with distractor suppression vs. distractor enhancement) modulate their selectivity across contexts.

*9. Page 14, first paragraph of Discussion section: In discussing the roles of prefrontal and posterior parietal cortex more broadly, the authors may wish to refer to Qi et al. (2015), J Neurophys 113(1):44-57.*

**Response:** We regret this omission; the paper by Qi et al., 2015 is now referenced in the relevant part of the Discussion.

*Reviewer #2 (Xiaojin Ma): This is an interesting study using single cell recording to pinpoint the neural activities in the FEF and LIP regions during singleton distractor suppression. As a disclaimer, my research involves electrophysiological techniques but unfortunately not single cell recording. Therefore, my comments are mainly on the appropriateness of experiment design to study attentional suppression, but not on the technical aspects unique to single cell recording or analysis.*

*The choice of the FEE and LIP do seem to be relevant to the study of attentional suppression. However, in the Introduction I hoped to see more explicit a priori hypotheses about how the activity patterns of the two regions would correspond to implications on different attributes of the mechanism of attentional suppression. This would pave the way for later interpreting differential neural responses in the two regions.*

**Response:** We thank the reviewer for her suggestion. We did formulate a hypothesis about the way salient stimuli could be encoded in parietal and prefrontal cortex in the second paragraph of the Introduction of the original manuscript: “It is thus possible that in prefrontal and parietal areas, salient distractors are encoded similarly to visual cortex, albeit by distinct subpopulations with specific functional properties”, which was largely confirmed by our results. Following the reviewer’s suggestion, we have extended this part of the Introduction to make the hypothesis and interpretation of such a possible result clearer: “Should this be the case, one could expect to find different groups of neurons in prefrontal (PFC) and/or parietal cortex; some encoding the salient stimulus through an early activity enhancement, other neurons showing a later suppression of activity for the salient distractor and possibly yet other neurons displaying an early enhancement followed by a later suppression. Such a finding would indicate that at the neuronal level, the early encoding of the salient distractor is not unique to purely visual areas. It is therefore still unclear whether and how neuronal subpopulations in areas that have been proposed to hold an attentional priority map, such as the Frontal Eye Fields (FEF) (Thompson et al., 2005) and the Lateral Intraparietal area (LIP) (Gottlieb et al., 1998), encode salient irrelevant stimuli during search*”.*

*The experiment had the target Identity change from trial to trial, which may have a few undesired effects. First, the frequent change in the top-down goal could possibly weaken the feature search mode that is necessary for suppression to occur. Second, the target on the 1-back trial may be a non-singleton distractor on the current trial. Intertrial priming of the target and the general selection history of every non-singleton shapes could inflate the target feature enhancement effect that was measured by saccades to the non-singleton. This may to an extent explain why the present study were able to find both suppression and enhancement effects in the two brain regions whereas the previous ones that used a fixed target identity only found suppression. I wonder what the consideration is behind the authors' decision to vary the target shape.*

**Response:** We thank the reviewer for bringing this up. The rationale behind our decision to vary the target from trial to trial was to make the task more naturalistic (i.e., in real life we do not always search for the same thing, nor do we get distracted by the exact same stimulus). Moreover, trials of the search tasks described herein, were presented to the monkeys in the same sessions with trials from a visual search task with various distractors of different colors and shapes in which we studied attentional priority based on feature similarity to the target (see Sapountzis et al., PNAS, 2018). The cueing conditions were the same (search for the target with a certain color and shape). Thus, the goal was to study how conspicuous silent distractors may be encoded in FEF and LIP under enhanced top-down input i.e. in subjects that are trained to search for a different stimulus on each trial close to what we do in our everyday life.

Note that the singleton present displays (Fig 1A) consisted of a red singleton in an array of green stimuli, a green singleton in an array of red stimuli, or a yellow singleton in an array of blue stimuli. The different configurations of the singleton present displays were randomly interleaved in each session, as well as with singleton absent (Fig 1B) or mixed-color displays (Fig 1C). Thus, it is not the case that the target in the previous trial was a non-singleton distractor in the current trial. In addition, we do not believe that this is the reason why we find both suppression and enhancement effects, whereas previous studies found only suppression. When averaging across the whole population (as was done in previous studies) we replicated previous studies’ findings by showing only suppression effects (Fig 2A). Enhancement effects became apparent only when we considered subpopulations of neurons, an approach not implemented in previous studies.

However, following the reviewer’s comment we now acknowledge this difference between our task design and that of previous studies. The last sentence of the sixth paragraph in the Discussion now reads “Another difference between our task and that of previous studies (Cosman et al, 2018; Ipata et al, 2006), is that in our task the target identity was not known in advance and changed from trial to trial. It is possible that in conditions where the target is always the same, a feature search mode is stronger, resulting in an overall more prominent suppression of distractors.”.

*As the authors mentioned in the paper, FEF and LIP are known for encoding saliency. It is thus important to distinguish in the results whether the decoded location information about the singleton reflect detection of its saliency or subsequent suppression of it utilizing its saliency signal. The latter was to an extent supported by the reduced firing rate to a singleton compared to a target in neural activity. The decoding results may also contain similar information, and it would be great to emphasize such distinction when interpreting the results. ERP studies have shown that the component suggesting suppression (PD) actually appears highly similar to the component suggesting saliency detection (Ppc), see a recent review paper on the PD component: Gaspelin et al (2023, J Cog Neuro).*

**Response:** Unfortunately, it is not possible to derive detection and suppression effects from the decoding analysis. The decoding analysis quantifies how distinguishable singleton and target are, based on activity across the neuronal population, and the algorithm works by taking into account both enhancement and suppression effects. However, clues about detection and suppression signals can be derived from the neuronal subpopulation analysis in Fig 4. There, we identified a cluster with an early singleton enhancement followed by singleton suppression (Cluster 2). This early enhancement could reflect a saliency detection signal. Interestingly, the latency of this enhancement was similar in both areas, suggesting an early involvement of both prefrontal and parietal cortex in singleton detection. By contrast, the suppression effect in Clusters 1 and 2 could reflect the attentional suppression of the singleton. How these signals relate to the PD and the Ppc components is indeed a very interesting question, and studies such as the one by Cosman et al., 2018 that combine invasive and ERP recordings are extremely useful in this regard. The paper by Gaspelin et al., 2023 is indeed very insightful and has been cited at several points in the paper.

Following this and a related comment by Reviewer #3, we have added one paragraph in the Discussion (fifth paragraph of the revised manuscript), which reads *“*Our results are consistent with the signal suppression hypothesis, which postulates that salient stimuli generate a bottom-up "attend-to-me" signal that competes for attention, but this early salience signal can be overridden by an inhibitory process that prevents attentional capture. Εvent-related potential (ERP) studies have suggested that the bottom-up salience signal is reflected by an early positivity posterior contralateral (Ppc) component that appears before the canonical P_D_ component, which indicates suppression (see Gaspelin & Kerzel in Gaspelin et al., 2023). Thus, it is possible that the early enhancement and later suppression effects found in our subpopulations are linked to the pre-attentive and active suppression mechanisms postulated by the signal suppression hypothesis. […]”.

*In the Method section, there mentioned 3 different target-distractor color combinations, but only two monkey served as subjects. I couldn't find information about how the colors were assigned/counterbalanced and whether the assignment remained constant throughout the task for each monkey. Please specify these important information. The Vatterott, Mozer, & Vercera (2018, AP&P) and Gaspelin & Luck (2018, JEPHPP) papers showed that fixed color assignments to both the target and the singleton are critical for suppression to consistently occur.*

**Response:** The number of monkeys that we used is typical for this type of experiments that require an extensive period of several months for training and data collection for each animal. Note, however, that the statistical inference is not based on the number of animals, but rather on the number of recorded neurons.

Indeed, three different target-distractor combinations were used in the singleton present displays (Fig 1A), which consisted of a red singleton in an array of green stimuli, a green singleton in an array of red stimuli, and a yellow singleton in an array of blue stimuli. The singleton present displays were randomly interleaved with each other, as well as with singleton absent (Fig 1B) or mixed-color (Fig 1C) displays in each session. All three color combinations and displays were presented to both monkeys. We are sorry if this was not clear. Following the reviewer’s comment we now clarify this in the Methods section.

The reviewer raises an interesting point by pointing to the papers by Gaspelin & Luck, 2018 and Vatterott, et al., 2018 who showed that familiarity with a repeating singleton can lead to its suppression within a block. More interestingly, however, the study by Vatterott, et al., 2018 showed that after extensive training with different singleton distractors, participants could effectively suppress novel color singletons without having previously experienced them. Thus, experience with different distractors and general task experience may allow participants to generalize their distractor rejection to novel singleton distractors. Additional research by Ma & Abrams, 2023 and Won, Kosoyan, & Geng, 2019 also provided evidence for second-order singleton suppression.

In our study, we did use fixed combinations of colors in the singleton displays for singleton and target/non singleton distractors. However, the color of the singleton distractor was not fixed. Critically, however, the singleton never served as the target during the entire training and recording sessions (which lasted several months), and as a result, the animals learned to effectively ignore singletons, as was evidenced by the significant behavioral and neuronal effects reported in the manuscript. Thus, we provide strong evidence that extensive experience with the task can lead to singleton distractor suppression. Following this and a relevant comment by Reviewer #3 (their fourth comment), we now discuss our results in the context of first- and second- order singleton suppression. The second paragraph of the Discussion now reads “Previous research has suggested that a color singleton can only be suppressed if its color is known in advance (i.e., first-order feature suppression; Gaspelin & Luck, JEP:HPP 2018). However, since the singleton color in our task was not known beforehand, our results show that experience with the task can lead to the suppression of a singleton regardless of its color (i.e., second-order feature suppression; Ma & Abrams, 2023; Won, Kosoyan, & Geng, 2019; Vatterott et al., 2017)”.

*References:*

*Gaspelin, N., Lamy, D., Egeth, H. E., Liesefeld, H. R., Kerzel, D., Mandal, A., ... & van Moorselaar, D. (2023). The distractor positivity component and the inhibition of distracting stimuli. Journal of cognitive neuroscience, 35(11), 1693-1715.*

*Vatterott, D. B., Mozer, M. C., & Vecera, S. P. (2018). Rejecting salient distractors: Generalization from experience. Attention, Perception, & Psychophysics, 80, 485-499.*

*Gaspelin, N., & Luck, S. J. (2018). Distinguishing among potential mechanisms of singleton suppression. Journal of Experimental Psychology: Human Perception and Performance, 44(4), 626.*

*Reviewer #3: Review of Sapountzis, Antoniadou, & Gregoriou, "Diverse activity profiles contribute to the control of distraction in the prefrontal and parietal cortex"*

*Manuscript ID: PBIOLOGY-D-24-02168R1*

*Summary*

*The authors tested whether salient color singleton distractors (e.g., a red item among all green items) are suppressed by the early visual system using recordings of rhesus monkeys in areas FEF and LIP. To test suppression, the authors presented the monkeys with singleton present trials and singleton absent trials with the task of fixating a specific-feature (e.g., a diamond shape) target among heterogeneously shaped distractors (e.g., stars, circles, etc.). Neuronal populations in FEF and LIP were recorded during the task and analyzed in many ways to support the overall notion that the color singleton distractors were suppressed, and this suppression cannot be explained by target feature enhancement. Further, the authors present evidence of different population types and areas of representation while also demonstrating the seemingly different roles of FEF and LIP in attentional selection.*

*Overall, the manuscript was a real pleasure to read! It was well written, easy to follow, and tests a fundamental and important question concerning distractor suppression and the neurobiology associated with it. The authors had clear predictions and logic. To the authors' credit, there were many claims tested and despite some of the complexities, the results were straightforward and surprisingly easy to interpret. I have a few critiques listed below, which to be clear, are meant to be (mostly) read as suggestions (with the aim of strengthening the connections to the literature), not precursors preventing publication.*

*Critique (in no particular order)*

1) *The authors test between target enhancement and distractor suppression, which was a welcome addition and a really cool finding (especially given the results suggest suppression). What might be potentially interesting would be to also relate these findings to recent claims about visual salience (e.g., Wang & Theeuwes, 2020 and the replies from Stilwell et al. 2023 & 2024). Briefly, the claim from stimulus-driven accounts was that the singletons were not salient enough to warrant suppression. Stilwell and colleagues developed methods to assess salience and in doing so, found evidence that the more salient the distractors became the greater the suppression (i.e., oculomotor suppression and probe-suppression). In the current study, it appears this may also be the case and would really complement recent support of the signal suppression hypothesis.* *The mixed color displays should render the singleton as less salient given less color contrast between the singleton and other items compared with the non-mixed displays. It appears that in Figure 3 the results support the claim that the more salient singleton (non-mixed displays) was suppressed more strongly than the mixed-color displays. Perhaps the authors could mention this finding and how it relates to signal suppression, especially recent claims of the role of salience.*

**Response:** We thank the reviewer for the positive feedback on the manuscript and constructive comments. Following the reviewer’s comment, we now discuss the debate on the relationship between the prominence of salience and the suppression of salient distractors in the context of our findings. The final sentences in the seventh paragraph of the Discussion now read *“*Our results also contribute to a recent debate in the literature on whether signal suppression occurs only with singletons that are not salient enough to capture attention (Wang & Theeuwes, 2020; Stilwell et al., 2023; Stilwell et al., 2024). Our finding that a salient singleton is more strongly suppressed in the FEF relative to the exact same stimulus presented in a non-salient context, provides additional support to the idea that the more salient the distractor, the stronger the suppression in agreement with previous studies (Stilwell et al., 2023; Stilwell et al., 2024)*”*

2) *In a similar vein to the previous comment, if you look at Figure 3C, for example, there also seems to be evidence that FEF might track the earlier saliency computations. Recently, there has been evidence that salient stimuli produce an EEG/ERP, Ppc component which has been argued to be a "saliency detector" (John McDonald and colleagues as well as Hermann Muller's group have been pushing this idea recently). In unpublished data from my own research, we manipulated the salience of the singleton distractor via color contrast and found a modulation of this early positivity (likely the Ppc) both in amplitude (~0.5 uV) and in timing (~100 ms post display onset) as a function of salience, prior to the canonical PD component indicative of suppression. The Ppc was both larger and earlier for the high- than low-salience singleton, yet the following PD component was the same. In the current study, especially in Figure 3C, it appears the FEF shows a reliable "blip" at approximately 100 ms for the AUROC analysis, followed by suppression. There also seem to be hints of this early (~100 ms) pattern in the firing rates of FEF as well (e.g., in Figure 3A the singleton distractor has a higher rate of firing at about 100 ms than the matched stimuli). This might support the idea that the early salience signals can be modulated (and reflected in changes to FEF populations), but the subsequent suppression is stronger for these more salient distractors. It seems like the responsive unit analyses (i.e., those depicted in Figure 4) seem to also support these notions (greater representation of the salient singleton early on). These might be fruitful analyses/discussion points to further cement the current findings in support of the signal suppression hypothesis and more recent findings regarding the role of salience, namely that there is pre-attentive salience processing (what used to be referred to as the "attend-to-me" signal) followed by active suppression (often termed "proactive" suppression, though it's better stated as prior to the first shift of attention).*

**Response:** We thank the reviewer for this comment. The reviewer is right that the early and late modulations seen in Figures 3 and 4 are consistent with the signal suppression hypothesis. It is possible that the early singleton enhancement in Fig 3 and in Cluster 2 of Fig 4B corresponds to a bottom-up saliency signal analogous to the early Ppc. Accordingly, both Cluster 1 and Cluster 2 (Fig 4B) show a singleton suppression effect that emerges later than the enhancement found in Cluster 2 (possibly corresponding to a top-down signal) and is evident earlier in the PFC. These results would indeed agree with the predictions of the signal suppression hypothesis. However, we wish to emphasize that the late singleton enhancement found in Cluster 3 may also contribute to the encoding of the singleton (see our population-level analysis), and perhaps to the mechanism that facilitates the suppression of irrelevant locations (although this may sound counterintuitive). Following the reviewer's comment, these points are now more explicitly discussed in a new paragraph in the Discussion (fifth paragraph), which reads: “Our results are consistent with the signal suppression hypothesis, which postulates that salient stimuli generate a bottom-up "attend-to-me" signal that competes for attention, but this early salience signal can be overridden by an inhibitory process that prevents attentional capture. Εvent-related potential (ERP) studies have suggested that the bottom-up salience signal is reflected by an early positivity posterior contralateral (Ppc) component that appears before the canonical P_D_ component, which indicates suppression (see Gaspelin & Kerzel in Gaspelin et al 2023). Thus, it is possible that the early enhancement and later suppression effects found in our subpopulations are linked to the pre-attentive and active suppression mechanisms postulated by the signal suppression hypothesis […]”.

*3) However, one thing to consider with the early enhancement followed by suppression results is the case of "rapid disengagement" which is argued by stimulus-driven accounts to explain all the suppression data (e.g., Theeuwes, 2010; 2024). The initial salience processing is selective in nature, and all suppression after this is "reactive" in nature. The clustering analyses seem to challenge if not refute the "early selection followed by rapid disengagement" idea given that some clusters show* *early suppression effects which seem plausibly too early for an initial shift of attention followed by rapid disengagement. Perhaps the authors could acknowledge this alternative and how the current study rules this out.*

**Response:** This is an interesting observation, and we thank the reviewer for this suggestion. We do believe that these enhancement and suppression effects contribute to a pre-attentive “priority map” that encodes the behavioral relevance of stimuli to guide visual search before they are overtly attended (e.g., Bichot et al., 2015; Sapountzis et al., 2018). However, it is not at all clear how early one would expect these effects to occur in the brain to be inconsistent with the rapid-disengagement account. Interestingly, Theeuwes, 2024 considers similar early enhancement followed by suppression effects in macaque V4 (Klink et al., 2023) to be consistent with rapid disengagement. Given that the latencies of suppression vary in the different subpopulations in our study and our latency estimates are not absolute, it is hard to postulate how early rapid disengagement should be reflected in the activity of different subpopulations. Interpreting neural effects as psychological processes should be done with caution. What the reviewer suggests is possible, but we believe that we cannot refute the rapid disengagement theory based on our results*.* It is, however, important for the community to establish clear predictions on how different processes associated with attentional capture would be reflected in neuronal activity.

Nevertheless, following the reviewer’s suggestion, we added a reference to the rapid disengagement hypothesis in the fifth paragraph of the discussion: “An initial transient enhancement in activity followed by suppression could also be consistent with the rapid disengagement theory, which assumes that attention is initially captured by the salient stimulus followed by rapid disengagement of attention from the salient distractor location (Theeuwes, 2010). Assessing whether the early activity enhancement in our study reflects attentional capture or rather merely low-level sensory properties associated with the singleton would require additional task conditions and was beyond the scope of this study.”

*4) The task used multiple singleton distractor colors which may have promoted "second-order" suppression (e.g., Gaspelin & Luck, 2018; Ma & Abrams, 2023; Won, Kosoyan, & Geng, 2019) in which case all of the salient singleton distractors were suppressed regardless of their specific colors. In the most recent version of the signal suppression hypothesis, the proponents argue the suppression is feature-specific (i.e., first-order in nature, e.g., Gaspelin & Luck, 2018, JEP:HPP). Perhaps the authors could briefly mention that the current patterns of suppression may be evidence for second- instead of first-order suppression.*

**Response:** We thank the reviewer for this important suggestion. Following this and a relevant comment by Reviewer #2 we have expanded the second paragraph of the Discussion to read “Previous research has suggested that a color singleton can only be suppressed if its color is known in advance (i.e., first-order feature suppression; Gaspelin & Luck, JEP:HPP 2018). However, since the singleton color in our task was not known beforehand, our results show that experience with the task can lead to the suppression of a singleton regardless of its color (i.e., second-order feature suppression; Ma & Abrams, 2023; Won, Kosoyan, & Geng, 2019; Vatterott et al., 2017)”.

*Minor points*

*1) Many of the figures with lines are difficult to see differences given the thickness of the lines. For example, in the figures depicting normalized firing rates (most Panel A's in the figures) it's difficult to discern (at least visually) where the conditions overlap and where they diverge. Also, the dashed lines have a lot of space between the dashes making it difficult to see the shape of the curve, perhaps the authors can decrease the dash spacing.*

**Response:** We thank the reviewer for this remark. Where necessary, we have made the lines thinner and reduced the dash spacing.

*2) Perhaps I missed this but why did the authors choose to analyze the time window from 150-200 ms, specifically? Is there an a priori reason to choose this window? For example, many (especially those in support of the signal suppression hypothesis using EEG/ERP) choose the start of the time window for the "early" PD component closer to 100 ms (~115-125 ms).*

**Response:** We chose this particular time window because this is when we see the most robust firing rate effects (e.g., Fig 2A, Fig 3A, Fig 4B, Fig 5A). It is possible that differences between tasks (e.g. overt vs covert tasks) affect the latency of these effects.
